# Comparison of residents’ approaches to clinical decisions before and after the implementation of Evidence Based Medicine course

**Published:** 2014-10

**Authors:** ZAHRA KARIMIAN, JAVAD KOJURI, MOHAMMAD MAHDI SAGHEB, ALI MAHBOUDI, MAHBOOBEH SABER, MITRA AMINI, MOHAMMAD REZA DEHGHANI

**Affiliations:** 1 Quality improvement in clinical education Research Center, Shiraz University of Medical Science, Shiraz, Iran;; 2 English Language Department, Paramedical School, Shiraz University of Medical Sciences, Shiraz, Iran

**Keywords:** Clinical, Evidence-based medicine, Decision making, Resident

## Abstract

**Introduction:** It has been found that the decision-making process in medicine is affected, to a large extent, by one’s experience, individual mentality, previous models, and common habitual approaches, in addition to scientific principles. Evidence-based medicine is an approach attempting to reinforce scientific, systematic and critical thinking in physicians and provide the ground for optimal decision making. In this connection, the purpose of the present study is to find out to what extent the education of evidence based medicine affects clinical decision making.

**Methods**: The present quasi-experimental study was carried out on 110 clinical residents, who started their education in September, 2012 and finally 62 residents filled out the questionnaires. The instrument used was a researcher-made questionnaire containing items on four decision-making approaches. The questionnaire was used both as a pre-test and a post-test to assess the residents’ viewpoints on decision making approaches. The validity of the questionnaire was determined using medical education and clinical professionals’ viewpoints, and the reliability was calculated through Chronbach alpha; it was found to be 0.93. The results were analyzed by paired t-test using SPSS, version 14.

**Results:** The results demonstrated that evidence-based medicine workshop significantly affected the residents’ decision-making approaches (p<0.001). The pre-test showed that principles-based, reference-based and routine model-based approaches were more preferred before the program (p<0.001). However, after the implementation of the program, the dominant approaches used by the residents in their decision making were evidence-based ones.

**Conclusion:** To develop the evidence-based approach, it is necessary for educational programs to continue steadily and goal-orientedly. In addition, the equipment infrastructure such as the Internet, access to data bases, scientific data, and clinical guides should develop more in the medical departments.

## Introduction

Physicians have to make decisions in their practice ranging from diagnosis to analysis and treatment of the disease. In the process of clinical decision making, a body of variables such as signs and symptoms, medical knowledge, prior experience, the models doctors have acquired from their professors and even conjectures, emotions and impulses can affect a physicians’ decision (1). Parallel to promotion and accreditation of clinical decisions, the concept of evidence-based medicine was a new and research oriented approach proposed by Guyatt, et al. at Mc Master University in Canada. The new trend has been welcomed and developed increasingly in medical schools all over the world (2-4). This approach in medicine means the integration of the physician’s clinical experiences with the best evidence and documentation or the proper application of the best objective evidence for making accurate, fair and known treatment and diagnostic decisions about the patients (5, 6). Evidence-based medicine tries to improve the quality of clinical decisions through developing and reinforcing the ability of raising questions, data search skills, critical evaluation, selection of the best evidence and documentation and the application of the results of critical analysis. Furthermore, it is an attempt to reduce the effects of errors arising from the subjective judgment, out-of-date data or uncritical and linear inferences through objective clinical decisions derived from reliable and up-to-date scientific evidence (7, 8).

With increasing volumes of medical data, students and scholars of this field more than ever need to retrieve the reliable and necessary data for diagnostic and treatment decisions from among large quantities of published articles. As a result, they should be able to criticize and analyze the reliability of the sources and the data. According to some evidence, most students search information from public Internet sites such as Google, and Wikipedia (9). However, they do not possess enough information about searching skills in scientific databases, advanced searching methods, collecting and refining the data and posing a variety of clinical questions. Therefore, in medical education, it is essential to develop the required skills of life-long, self-directed learning, and the ability of posing questions in medical students (10).

Iranian medical universities have also adopted evidence-based medicine as a new approach in medical education. A variety of workshops have been held to develop the concept and philosophy of evidence-based medicine throughout the country. In the Iranian comprehensive health map, change in health education system and delivery of services by those who are knowledgeable, competent and accountable to the needs of the society is stressed (11).

Although different clinical groups including professors, clinical residents, medical students and other medical and paramedical staff play a part in development of evidence-based medicine, the role of clinical residents is of critical importance, because they not only deliver specialized clinical services, but also have a direct influence on the education and model adoption of junior residents and students. It has been revealed that medical students receive most of their information through interaction with clinical residents more than the time they spend with full-time faculty members (12, 13).

The term “resident” or medical specialty student implies living in the hospital. Rider, et al. call medical residents “hospital instructors” (14). Therefore, clinical residents are valuable, potential sources of education who, because of their close contact with junior students, can teach the most necessary, practical, clinical, and educational points (15, 16). Numerous studies, for example, have demonstrated that senior residents spend a lot of their time teaching junior residents and medical students (17-20). Medical students have pointed out that over one-third of their learning in clinical settings has been made by residents (21) and have considered the residents’ role, particularly during the first year on the clinical wards, as critical and determining (22-24).

According to Sánchez et al.’s findings at Mexico National University, senior residents maintained that they spend more than 32.5% of their time teaching medical and paramedical students and junior residents. Sánchez believes that universities of medical sciences should assess the residents’ educational needs and arrange well to meet them (25). In addition, the experience of Kathmandu University indicated that life-long learning, active and self-directed learning and evidence-based medicine are essential to clinical residents (26).

Regarding the model role of clinical residents in development of evidence-based education in junior students, identifying their treatment methods and clinical decisions is very significant in both patients’ health and medical education. Therefore, the present study aimed to investigate the clinical decision making by clinical residents of Shiraz University of Medical Sciences before and after implementing evidence-based medicine. 

## Methods

The present quasi-experimental study was carried out using a researcher-made questionnaire in 2013. The participants comprised all of the 110 clinical residents at Shiraz University of Medical Sciences, entering the university in October, 2012. They took part in a 30 hour educational program over five days in a row. The contents of the program included the main topics of evidence-based medicine to the suggested content by Ministry of Health and Treatment. The method of education was planned based on active learning and the five steps of evidence-based medicine. 

In all steps, the main approach of education was planned and implemented based on active learning, raising advanced clinical questions, searching data sources, assessing the retrieved articles, selecting options, final decision making and ultimately assessing of performance. In order to find out the residents’ viewpoints on decision making methods, we used a questionnaire containing two sections. To prepare the questionnaire, we first raised an open ended question: “How are the clinical decisions usually made on wards?” A specialized focal group including 10 faculty members with pediatrics, social medicine, cardiology, nephrology, neurology, surgery, medical education, gastroenterology, and gynecology specialties answered the question. Having summarized the responses, the researchers came up with 10 decision making approaches, forming the main items of the questionnaire. The items were categorized into four general topics including “scientific principles”, “Evidence-Based Medicine approach”, “Subjective Personal Experiences”, and “Modeling”.

The rate of using decision-making methods was calculated using Likert-scale scoring system, i.e. “rarely=1”, “sometimes=2”, and “usually=3” before and after the implementation of the program. The second section of the questionnaire included eight items as complementary data about prerequisites of evidence-based medicine. Five medical education professionals with clinical specialty helped to prepare the items.

One month after the program, the questionnaires were sent to the clinical wards. To observe the ethical considerations, the questionnaires were filled out and collected anonymously. Of the 110 questionnaires sent, 62 were completed fully and precisely and returned. For descriptive statistics, frequency and standard deviation were calculated and for the inferential statistics, Paired sample t-test and Independent sample t-test were used through SPSS, version 14 (SPSS Inc, Chicago, IL, USA). 

## Results

Of the 62 participants of the study, 19 (%31.7) were male, and 41 (%68.3) female. The age range of the participants was from 25 to 40 with a mean of 31.1±4.1. The results showed that the evidence-based medicine training program affected the residents’ decision making significantly. Furthermore, %53 of the residents asserted, after the program, that they used evidence-based medicine approach in teaching junior residents and medical students to a great extent. They also stated that they encouraged the students to make use of this approach. Table 1 displays the frequency of the participants’ viewpoints on each category.

**Table 1:** The frequency of decision making approaches used by residents

**Decision making approach**		**Before the program**			**After the program**		
		**Usually**	**Sometimes**	**Rarely**	**Usually**	**Sometimes**	**Rarely**
**Scientific principle**	Consulting references books	43(70.5)	17(27.9)	1(1.6)	37(64.9)	20(35.1)	0(0)
	Using abridged pocket Books	13(21)	23(37.1)	26(41.9)	13(21.3)	23(37.7)	25(41.1)
**Evidence based** **medicine**	Searching article in internet	24(40)	28(46.7)	8(13.3)	20(33.9)	28(47.5)	11(18.6)
	Using ward guidelines	10(16.7)	21(35)	29(48.3)	10(16.6)	25(41.7)	25(41.7)
	Critical appraisal article and evidence	4(6.7)	14(23.3)	42(70)	9(14.5)	45(72.6)	8(12.9)
**Subjective personal experiences**	Using personal experiences	42.4% (25)	44.1% (26)	13.6% (8)	33.9% (19)	42.9% (24)	23.2% (13)
	Subjective and rapid judgment	16(25.8)	31(50)	15(24.2)	6(9.8)	32(52.5)	23(37.7)
	Consulting with or asking from coworkers	19(30.6)	37(59.7)	6(9.7)	15(24.6)	35(57.4)	11(18)
**Modeling**	Using professor’s methods	35(57.4)	23(37.7)	3(4.9)	26(43.3)	28(46.7)	6(10)
	Using clinical wards’ routines	19(31.7)	8(55)	43(13.3)	43(20)	34(56.7)	14(23.3)

**Figure 1:**
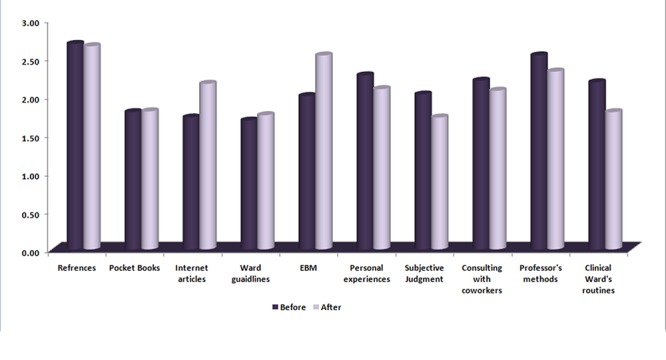
Comparison of decision making approaches by residents in clinical wards

The mean scores of the residents’ viewpoints on decision making approaches before and after the training program are shown in Figure 1.

The results of T-paired test showed that residents’ clinical decision making using evidence-based approach improved significantly in comparison to that before the training program (p<0.001). Furthermore, decision making approaches based on “personal judgment and experience” and “modeling” decreased in comparison to the use of the methods before the program (p<0.001). However, the use of logical and sensible methods such as consulting scientific references or pocket guidebooks did not change significantly (p=0.52)(Table 2).

**Table 2:** Analytic comparison of decision making approaches by residents in clinical wards

**Decision making methods**		**Mean±SD**	**df**	**t**	**p **
**Scientific principle**	Before	2.24±0.43	60	0.637	0.520
	After	2.19±0.49			
**Evidence based medicine**	Before	1.59±0.50	61	6.66	<0.001
	After	1.97±0.46			
**Subjective personal experiences**	Before	2.16±0.49	61	4.04	<0.001
	After	1.96±0.46			
**Modeling**	Before	2.37±0.46	61	3.60	<0.001
	After	2.14±0.49			

Analyzing the prerequisite skills for the application of evidence-based medicine, we found that about 43% of the residents most often had enough skills to search the required articles. 16.6 percent of the residents could most often and 67.2 percent of them could sometimes download their required articles. 77.4 percent of the participants most often had access to the Internet. Only 18 percent of the residents did have the experience of publishing a research article. Also, only 22.6 percent of the residents did state that the computers in the ward were equipped with up-to-date CDs (as an up-to-date source of scientific articles). And only 37.2 percent of them did say that there were clinical guidelines on their wards. 

The residents were asked an open-ended question: “what sites and databases did you use to search articles?” before and after the training program. Most residents answered that they usually used general sites such as Google or Yahoo before the program but after the program they got familiar with specialized databases to some extent. 

## Discussion

Experts believe that evidence-based medicine is an approach which can reinforce systematic, critical and scientific thinking and provide the ground for optimal clinical decisions. Decision making, selection and application of documentary evidence are the main parts of evidence-based medicine because this can finally affect the patients’ health (15, 27, 28) According to the findings of the present study, two methods of “reliance on established scientific principles” (2.24) and “model making from the environment” (2.37) were the most preferred methods used by residents in their decision-making (2.24). Although the use of logical and reasonable methods such as “use of reference books” was relatively predictable because of their scientific and model-oriented nature of medicine, the findings demonstrated that the use of methods such as “subjective judgment based on personal experience” was as frequent as the use of “logical and reasonable” methods among the residents (2.16). This is both clinically and educationally interesting. The least used method before the training program was the method based on documentary and critical thinking (1.59). Sadeghi et al. in their study (2011) found out that the most common sources for residents in their decision making were the “use of reference books” (%59.6), “clinical experiences” (%44), and only 19 percent of the residents used new articles (29).

Comparing the use of decision making approaches after the program, we found that the training course significantly affected the residents’ use of decision making approaches, or the training program at least directed them to more proper decision making approaches. Although there was no significant difference between the use of logical and sensible methods based on scientific approaches before and after the training program, the decision making approaches based on experience and personal judgment as well as getting model from the environment, which are not scientifically based and may merely arise from habituation and observations, were significantly used less often after the training. Furthermore, the use of evidence-based approach increased significantly after the training. The residents stated that they made use of this approach in teaching undergraduates.

A lot of studies have demonstrated the positive effects of evidence-based medicine on residents’ attitudes, knowledge, and skills (30-36). Such an approach should not only be maintained but also reinforced in clinical settings. Clinical residents, who possess the highest and most important academic degrees, require research competence and attitude, while based on the findings of this study only 16 percent of the residents taking part in this study had some experience regarding the publication of articles.

Research activities are in close connection with evidence-based medicine as they follow the scientific approach, from asking questions to looking for and studying scientific sources to organized methodology to presenting results to critical thinking and finally to knowledge publication. As a result, it is necessary to include the required courses in the general medicine curriculum to reinforce the students’ thinking and research skills. This can provide a ground for further development in postgraduate studies. The science-oriented research approach is currently used in most universities throughout the world. Wadland et al. (1999) have stressed the improvement of proposal writing and research skills for medical students. Barnet et al. (2000) and Dorsch et al. (2004) consider evidence-based approach as the basis of life-long learning (30, 31, 35). West et al. (2011) have emphsized the education of basic skills of epidemiology and biostatistics in medical curriculum (36). By and large, training on evidence-based medicine has increased the residents’ attitude towards decision making approaches and their application. This makes proper planning for this trend a necessity. The basis should be put during the general medicine curriculum and then the training should be extended through residency study period.

## Conclusion

Overall, it seems that training the EBM courses has had a positive influence on the residents' approach to the use of scientific evidence and recommended to continue the EBM courses for other residents. Also, for further development and reinforcement of evidence-based approach in clinical decision making, the provision of infrastructures such as the Internet and accessible databases, scientific gatherings among residents such as journal clubs on evidence-based medicine and compiling educational guidelines in clinical departments are necessary. 

## References

[1] Mortaz-Hajari S, Davani-Zadeh M, Soltani A (2009). principles of clinical reasoning and decision making.

[2] Glasziou P, Del Mar C, Salisbury J (2010). Evidence based medicine workbook.

[3] Irani S, Soltani A (2009). Critical appraisal of RSTs and case series.

[4] Sackett DL, Rosenberg WM, Gray J, Haynes RB, Richardson WS (1996). Evidence based medicine: what it is and what it isn't. BMJ.

[5] Dickersin K, Straus SE, Bero LA (2007). Evidence based medicine: increasing, not dictating, choice. BMJ.

[6] Greenhalgh T, Donald A (2000). Evidence based health care workbook.

[7] Finkel ML, Brown HA, Gerber LM, Supino PG (2003). Teaching evidence-based medicine to medical students. Med Teach.

[8] Montori VM, Guyatt GH (2008). Progress in evidence-based medicine. JAMA.

[9] Mi M (2012). Evidence based medicine teaching in undergraduate medical education: a literature review. Evidence based library and information practice.

[10] Glasziou P, Burls A, Gilbert R (2008). Evidence based medicine and the medical curriculum. BMJ (Clinical research ed).

[11] (2009). Comperehensive Health Map [Internet].

[12] Mann KV, Sutton E, Frank B (2007). Twelve tips for preparing residents as teachers. Med Teach.

[13] Spickard ΙΙΙA, Corbett JR, Schorling JB (1996). Improving residents’ teaching skills and attitudes toward teaching. Journal of general internal medicine.

[14] Rider E, Federman D, Hafler J (2000). Residents as teachers-a faculty development approach to programme development. Med Educ.

[15] Busari JO, Scherpbier AJ, Van Der Vleuten CP, Essed GG (2003). The perceptions of attending doctors of the role of residents as teachers of undergraduate clinical students. Med Educ.

[16] Wilson FC (2007). Teaching by residents. Clinical orthopaedics and related research.

[17] Armstrong E, Ashford I, Freeman J, Koller C, Pistoria M, Stello B (2001). Developing The Teaching Skills of Residents Through Interactive Resident-as-Teacher Workshops.

[18] Brown RS (1970). House staff attitudes toward teaching. Acad Med.

[19] Edwards JC, Friedland JA (2002). Residents' teaching skills.

[20] Wilkerson L, Lesky L, Medio FJ (1986). The resident as teacher during work rounds. Acad Med.

[21] Barrow M (1966). Medical Students Opinions of the House Office as a Medical Educator. Med Educ.

[22] Bing-You RG, Sproul MS (1992). Medical students' perceptions of themselves and residents as teachers. Med Teach.

[23] Morrison EH, Hollingshead J, Hubbell FA, Hitchcock MA, Rucker L, Prislin MD (2002). Reach Out and Teach Som eone: Generalist Residen ts’ Needs for Teaching Skills Development. Fam Med.

[24] Whittaker JR, Estes NC, Ash J, Meyer LE (2006). The value of resident teaching to improve student perceptions of surgery clerkships and surgical career choices. The American journal of surgery.

[25] Sánchez-Mendiola M, Graue-Wiechers EL, Ruiz-Pérez LC, García-Durán R, Durante-Montiel I (2010). The resident-as-teacher educational challenge: a needs assessment survey at the National Autonomous University of Mexico Faculty of Medicine. BMC medical education.

[26] Bhattarai M (2007). Study skills course in medical education for postgraduate residents. Kathmandu University Medical Journal.

[27] Heneghan C, Badenoch D (2008). Evidence-based medicine toolkit.

[28] Nordenstrom J (2008). Evidence-Based Medicine: In Sherlock Holmes' Footsteps.

[29] Sadeghi M, Khanjani N, Motamedi F (2011). Knowledge, attitude and application of evidence based medicine (EBM) among residents of Kerman Medical Sciences University. Iranian Journal of Epidemiology.

[30] Barnett SH, Kaiser S, Morgan LK, Sullivant J, Siu A, Rose D (2000). An integrated program for evidence-based medicine in medical school. The Mount Sinai journal of medicine, New York.

[31] Dorsch JL, Aiyer MK, Meyer LE (2004). Impact of an evidence-based medicine curriculum on medical students' attitudes and skills. Journal of the Medical Library Association.

[32] Ghali WA, Saitz R, Eskew AH, Gupta M, Quan H, Hershman WY (2000). Successful teaching in evidence‐based medicine. Med Educ.

[33] Holloway R, Nesbit K, Bordley D, Noyes K (2004). Teaching and evaluating first and second year medical students' practice of evidence‐based medicine. Med Educ.

[34] Schilling K, Wiecha J, Polineni D, Khalil S (2006). An interactive Web-based curriculum on evidence-based medicine: Design and effectiveness. Family medicine-kansas city.

[35] Wadland WC, Barry HC, Farquhar L, Holzman C, White A (1999). Training medical students in evidence-based medicine: a community campus approach. Family medicine-kansas city.

[36] West CP, Jaeger TM, McDonald FS (2011). Extended evaluation of a longitudinal medical school evidence-based medicine curriculum. Journal of general internal medicine.

